# Measuring the functional sequence complexity of proteins

**DOI:** 10.1186/1742-4682-4-47

**Published:** 2007-12-06

**Authors:** Kirk K Durston, David KY Chiu, David L Abel, Jack T Trevors

**Affiliations:** 1 Department of Biophysics, University of Guelph, Guelph, ON, N1G 2W1, Canada; 2 Department of Computing and Information Science, University of Guelph, Guelph, ON, N1G 2W1, Canada; 3 Program Director, The Gene Emergence Project, The Origin-of-Life Foundation, Inc., 113 Hedgewood Drive Greenbelt, MD 20770-1610, USA; 4 Department of Environmental Biology, University of Guelph, Guelph, ON, N1G 2W1, Canada

## Abstract

**Background:**

Abel and Trevors have delineated three aspects of sequence complexity, Random Sequence Complexity (RSC), Ordered Sequence Complexity (OSC) and Functional Sequence Complexity (FSC) observed in biosequences such as proteins. In this paper, we provide a method to measure functional sequence complexity.

**Methods and Results:**

We have extended Shannon uncertainty by incorporating the data variable with a functionality variable. The resulting measured unit, which we call Functional bit (Fit), is calculated from the sequence data jointly with the defined functionality variable. To demonstrate the relevance to functional bioinformatics, a method to measure functional sequence complexity was developed and applied to 35 protein families. Considerations were made in determining how the measure can be used to correlate functionality when relating to the whole molecule and sub-molecule. In the experiment, we show that when the proposed measure is applied to the aligned protein sequences of ubiquitin, 6 of the 7 highest value sites correlate with the binding domain.

**Conclusion:**

For future extensions, measures of functional bioinformatics may provide a means to evaluate potential evolving pathways from effects such as mutations, as well as analyzing the internal structural and functional relationships within the 3-D structure of proteins.

## Background

There has been increasing recognition that genes deal with information processing. They have been referred to as "subroutines within a much larger operating system". For this reason, approaches previously reserved for computer science are now increasingly being applied to computational biology [1]. If genes can be thought of as information-processing subroutines, then proteins can be analyzed in terms of the products of information interacting with laws of physics. It may be possible to advance our knowledge of proteins, such as their structure and functions, by examining the patterns of functional information when studying a protein family.

Our proposed method is based on mathematical and computational concepts (e.g., measures). We show here that, at least in some cases in sequence analysis, the proposed measure is useful in analyzing protein families with interpretable experimental results.

Abel and Trevors have delineated three qualitative aspects of linear digital sequence complexity [2,3], Random Sequence Complexity (RSC), Ordered Sequence Complexity (OSC) and Functional Sequence Complexity (FSC). RSC corresponds to stochastic ensembles with minimal physicochemical bias and little or no tendency toward functional free-energy binding. OSC is usually patterned either by the natural regularities described by physical laws or by statistically weighted means. For example, a physico-chemical self-ordering tendency creates redundant patterns such as highly-patterned polysaccharides and the polyadenosines adsorbed onto montmorillonite [4]. Repeating motifs, with or without biofunction, result in observed OSC in nucleic acid sequences. The redundancy in OSC can, in principle, be compressed by an algorithm shorter than the sequence itself. As Abel and Trevors have pointed out, neither RSC nor OSC, or any combination of the two, is sufficient to describe the functional complexity observed in living organisms, for neither includes the additional dimension of functionality, which is essential for life [5]. FSC includes the dimension of functionality [2,3]. Szostak [6] argued that neither Shannon's original measure of uncertainty [7] nor the measure of algorithmic complexity [8] are sufficient. Shannon's classical information theory does not consider the meaning, or function, of a message. Algorithmic complexity fails to account for the observation that 'different molecular structures may be functionally equivalent'. For this reason, Szostak suggested that a new measure of information–functional information–is required [6]. Chiu, Wong, and Cheung also discussed the insufficiency of Shannon uncertainty [9,10] when applied to measuring outcomes of variables. The differences between RSC, OSC and FSC in living organisms are necessary and useful in describing biosequences of living organisms.

Consider two main uses for the proposed method for measuring FSC, which incorporates functionality: 1) comparative analysis of biosequence subgroups when explicit time lag is known; and 2) typicality analysis between biosequence subgroups when there is no explicit time lag. In the first case, such as an evolutionary time scale, an increase or decrease in FSC between an earlier gene or protein and a later gene or protein can be measured, evaluating its possible degradations and/or functional effects due to various changes such as insertions, deletions, mutations and rearrangements. In the second case, a large set of aligned sequences representing a protein family can be subdivided according to phylogenetic relationships derived from typicality of species groupings and the FSC for each subgroup measured. This is important when evaluating the emergence or evolution of viral or microbial strains with novel functions such as in the comparisons of the Chlamydia family genomes [11]. An analysis may reveal the extent of the difference in FSC between one functional group and the other, as well as the modular interactions of the internal relationship structure of the sequences [12].

The ability to measure FSC would be a significant advance in the ability to identify, analyze, compare, and predict the metabolic utility of biopolymeric sequences. Mutational drift, emerging pathogenic viral and microbial species/strains, generated mutations, acquired heritable diseases and mutagenic effects could all be evaluated quantitatively. Furthermore, *In vitro *experiments using SELEX [13-15] to study transitions in possible early ribozyme family growth could then be evaluated in a quantitative, as well as qualitative and intuitive fashion. Evolutionary changes, both actual and theoretical, can also be evaluated using FSC.

It is known that the variability of data can be measured using Shannon uncertainty [16]. However, Shannon's original formulation when applied to biological sequences does not express variations related to biological functionality such as metabolic utility. Shannon uncertainty, however, can be extended to measure *the joint variable *(*X, F*), where *X *represents the variability of data, and *F *functionality. This explicitly incorporates empirical knowledge of metabolic function into the measure that is usually important for evaluating sequence complexity. This measure of both the observed data and a conceptual variable of function jointly can be called *Functional Uncertainty *(*H*_*f*_) [17], and is defined by the equation:

*H*(*X*_f_(*t*)) = -∑*P*(*X*_f_(*t*)) log*P*(*X*_f_(*t*))

where *X*_f _denotes the conditional variable of the given sequence data (*X*) on the described biological function *f *which is an outcome of the variable (*F*). For example, a set of 2,442 aligned sequences of proteins belonging to the ubiquitin protein family (used in the experiment later) can be assumed to satisfy the same specified function *f*, where *f *might represent the known 3-D structure of the ubiquitin protein family, or some other function common to ubiquitin. The entire set of aligned sequences that satisfies that function, therefore, constitutes the outcomes of *X*_f_. Here, functionality relates to the whole protein family which can be inputted from a database. The advantage of using *H*(*X*_f_(*t*)) is that changes in the functionality characteristics can be incorporated and analyzed. Furthermore, the data can be a single monomer, or a biosequence, or an entire set of aligned sequences all having the same common function. The significance of the statistical variations can then be evaluated if necessary [8]. The state variable *t*, representing time or a sequence of ordered events, can be fixed, discrete, or continuous. Discrete changes may be represented as discrete time states.

Functional bioinformatics is emerging as an important area of research [18-20]. Even though the term 'biological function' has been freely used for specific experimentation, there is no generally consistent usage of the term. According to Karp [21], biological functionality can refer to biochemical specified reactions, cellular responses, and structural properties of proteins and nucleic acids. It can be defined or specified at the global level (i.e., the entire organism), locally at the sub-molecular level, or applicable to the whole molecule. Hence confusion exists in interpreting its meaning. Karp [21] recognized that biological function is a complex concept. Using Webster, he refers to 'specially fitted' action or 'normal and specific contribution' of a part to the economy of the whole. Function can be related to cellular components (e.g., macromolecules, proteins or small molecules) that interact and catalyze biochemical transformations. In specific applications, it can be a local function of an enzyme such as the substrate that is acted on, or the ligands that activate or inhibit the enzyme. In more systemic integrated functions, it may refer to pathways, single or multiple, in a hierarchical, nested scope [22,23]. In general, it is a challenge 'to define a single best set of biologically acceptable rules for performing this decomposition' [21].

In our approach, we leave the specific defined meaning of functionality as an input to the application, in reference to the whole sequence family. It may represent a particular domain, or the whole protein structure, or any specified function with respect to the cell. Mathematically, it is defined precisely as an outcome of a discrete-valued variable, denoted as *F=*{*f*}. The set of outcomes can be thought of as specified biological states. They are presumed non-overlapping, but can be extended to be fuzzy elements. In order to get a meaningful calculation in measuring FSC, the measure should be statistically significant in practice [7,10], much larger than zero when relating to the sequences. When sequences are chosen that are unrelated to the function to be analyzed, or are simply arbitrarily ordered or randomly generated sequences, then the measure of FSC will be small and statistically not significant. For example, if many sequences that do not share the same function *f*, are mistakenly included within an aligned set representing some particular function, we should expect the measure of FSC of that set to be degraded, possibly even to a very small value. However, when the specified functionality is chosen meaningfully (even in part), then FSC can be interpreted.

Consider further, when a biosequence is mutated, the mutating sequence can be compared at two different time states going from *t*_i _to *t*_j_. For example, *t*_i _could represent an ancestral gene and *t*_j _a current mutant allele. Different sequences sharing the same function *f *(as outcomes of the variables denoted respectively as *X*_f_, *Y*_f_) can also be compared at the same time *t*. Within a sequence, any change of a monomer in the sequence represents a single step change that may or may not affect the overall function. Sequence reversals, gene splits, lateral transfers, and multiple point mutations can also be quantified between the two states *t*_i_, *t*_j_. The limits of the change in functional uncertainty between the two states can then be evaluated at *t *= *t*_i _and *t *= *t*_*j*_.

The change in functional uncertainty (denoted as Δ*H*_f_) between two states can be defined as

Δ*H *(*X*_g_(*t*_i_), *X*_f_(*t*_j_)) = *H*(*X*_g_(*t*_j_)) - *H*(*X*_f_(*t*_i_))

where *X*_f _(*t*_i_) and *X*_g _(*t*_j_) can be applied to the same sequence at two different times or to two different sequences at the same time. Δ*H*_f _*can then quantify the change in functional uncertainty between two biopolymeric states with regard to biological functionality*. Unrelated biomolecules with the same function or the same sequence evolving a new or additional function through genetic drift can be compared and analyzed. A measure of Δ*H*_f _can increase, decrease, or remain unchanged.

Biological function is mostly, though not entirely determined by the organism's genetic instructions [24-26]. The function could theoretically arise stochastically through mutational changes coupled with selection pressure, or through human experimenter involvement [13-15]. A time limit can be set in some situations to evaluate what changes to *X*_f_(*t*_i_) might be possible within that limit. For example, an estimation of the evolutionary limits projected over the next 10 years could be computed in this approach for any particular strain of the HIV virus. The specifics of the function (as an outcome of the function variable) can remain constant, or it can be permitted to vary within a range of efficiency. The limit may be determined by what is permitted metabolically. There is often a minimum limit of catalytic efficiency required by the organism for a given function.

The *ground state g *(an outcome of *F*) of a system is the state of presumed highest uncertainty (not necessarily equally probable) permitted by the constraints of the physical system, when no specified biological function is required or present. Certain physical systems may constrain the number of options in the ground state so that not all possible sequences are equally probable [27]. An example of a highly constrained ground state resulting in a highly ordered sequence occurs when the phosphorimidazolide of adenosine is added daily to a decameric primer bound to montmorillonite clay, producing a perfectly ordered, 50-mer sequence of polyadenosine [3]. In this case, the ground state permits only one single possible sequence. Since the ground state represents the state of presumed highest uncertainty permitted by the physical constraints of the system, the set of functional options, if there are any, will therefore be a subset of the permitted options, assuming the constraints for the physical system remain constant. If the ground state permits only one sequence, then there is no possibility of change in the functional uncertainty of the system.

The *null state*, a possible outcome of *F *denoted as ø, is defined here as a special case of the ground state of highest uncertainly when the physical system imposes *no constraints at all*, *resulting in the equi-probability of all possible sequences or options*. Such sequencing has been called "dynamically inert, dynamically decoupled, or dynamically incoherent" [28,29]. For example, the ground state of a 300 amino acid protein family can be represented by a completely random 300 amino acid sequence where functional constraints have been loosened such that any of the 20 amino acids will suffice at any of the 300 sites. From Eqn. (1) the functional uncertainty of the null state is represented as

*H*(*X*_ø_(*t*_i_))= - ∑*P*(*X*_ø_(*t*_i_)) log *P*(*X*_ø_(*t*_i_))

where (*X*_ø_(*t*_i_)) is the conditional variable for all possible equiprobable sequences. Consider the number of all possible sequences is denoted by *W*. Letting the length of each sequence be denoted by *N *and the number of possible options at each site in the sequence be denoted by *m*, *W *= *m*^*N*^. For example, for a protein of length *N *= 257 and assuming that the number of possible options at each site is *m *= 20, *W *= 20^257^. Since, for the null state, we are requiring that there are no constraints and all possible sequences are equally probable, *P*(*X*_ø_(*t*_i_)) = 1/*W *and

*H*(*X*_ø_(*t*_i_))= - ∑(1/*W*) log (1/*W*) = log *W*.

The change in functional uncertainty from the null state is, therefore,

Δ*H*(*X*_ø_(*t*_i_), *X*_f_(*t*_j_)) = log (*W*) - *H*(*X*_f_(*t*_i_)).

Physical constraints increase order and change the ground state away from the null state, restricting freedom of selection and reducing functional sequencing possibilities, as mentioned earlier. The genetic code, for example, makes the synthesis and use of certain amino acids more probable than others, which could influence the ground state for proteins. However, for proteins, the data indicates that, although amino acids may naturally form a nonrandom sequence when polymerized in a dilute solution of amino acids [30], actual dipeptide frequencies and single nucleotide frequencies in proteins are closer to random than ordered [31]. For this reason, the ground state for biosequences can be approximated by the null state. The value for the measured FSC of protein motifs can be calculated by relating the joint (*X*, *F*) pattern to a stochastic ensemble, the null state in the case of biopolymers that includes any random string from the sequence space.

### A. Functional uncertainty as a measure of FSC

The measure of Functional Sequence Complexity, denoted as ζ, is defined as the change in functional uncertainty from the ground state *H*(*X*_g_(*t*_i_)) to the functional state *H*(*X*_f_(*t*_i_)), or

ζ = Δ*H *(*X*_g_(*t*_i_), *X*_f_(*t*_j_)).

The resulting unit of measure is defined on the joint data and functionality variable, which we call *Fits (*or *F*unctional *bits)*. The unit Fit thus defined is related to the intuitive concept of *functional *information, including genetic instruction and, thus, provides an important distinction between functional information and Shannon information [6,32].

Eqn. (6) describes a measure to calculate the functional information of the whole molecule, that is, with respect to the functionality of the protein considered. The functionality of the protein can be known and is consistent with the whole protein family, given as inputs from the database. However, the functionality of a sub-sequence or particular sites of a molecule can be substantially different [12]. The functionality of a sub-molecule, though clearly extremely important, has to be identified and discovered. This problem of estimating the functionality as well as where it is expressed at the sub-molecular level is currently an active area of research in our group.

To avoid the complication of considering functionality at the sub-molecular level, we crudely assume that each site in a molecule, when calculated to have a high measure of FSC, correlates with the functionality of the whole molecule. The measure of FSC of the whole molecule, is then the total sum of the measured FSC for each site in the aligned sequences.

Consider that there are usually only 20 different amino acids possible per site for proteins, Eqn. (6) can be used to calculate a maximum Fit value/protein amino acid site of 4.32 Fits/site. We use the formula log (*20*) - *H*(*X*_f_) to calculate the functional information at a site specified by the variable *X*_f _such that *X*_f _corresponds to the aligned amino acids of each sequence with the same molecular function *f*. The measured FSC for the whole protein is then calculated as the summation of that for all aligned sites. The number of Fits quantifies the degree of algorithmic challenge, in terms of probability, in achieving needed metabolic function. For example, if we find that the Ribosomal S12 protein family has a Fit value of 379, we can use the equations presented thus far to predict that there are about 10^49 ^different 121-residue sequences that could fall into the Ribsomal S12 family of proteins, resulting in an evolutionary search target of approximately 10^-106 ^percent of 121-residue sequence space. In general, the higher the Fit value, the more functional information is required to encode the particular function in order to find it in sequence space. A high Fit value for individual sites within a protein indicates sites that require a high degree of functional information. High Fit values may also point to the key structural or binding sites within the overall 3-D structure. Since the functional uncertainty, as defined by Eqn. (1) is proportional to the -log of the probability, we can see that the cost of a linear increase in FSC is an exponential decrease in probability.

For the current approach, both equi-probability of monomer availability/reactivity and independence of selection at each site within the strand can be assumed as a starting point, using the null state as our ground state. For the functional state, however, an *a posteriori *probability estimate based on the given aligned sequence ensemble must be made. Although there are a variety of methods to estimate *P*(*X*_f_(*t*)), the method we use here, as an approximation, is as follows. First, a set of aligned sequences with the same presumed function, is produced by methods such as CLUSTAL, downloaded from Pfam. Since real sequence data is used, the effect of the genetic code on amino acid frequency is already incorporated into the outcome. Let the total number of sequences with the specified function in the set be denoted by *M*. The data set can be represented by the N-tuple *X *= (*X*_1_, ... *X*_N_) where *N *denotes the aligned sequence length as mentioned earlier. The total number of occurrences, denoted by *d*, of a specific amino acid "aa" in a given site is computed. An estimate for the probability that the given amino acid will occur in that site *X*_*i*_, denoted by *P*(*X*_*i *_= "aa") is then made by dividing the number of occurrences *d *by *M*, or,

*P*(*X*_*i *_= "aa") = *d*/*M*.

For example, if in a set of 2,134 aligned sequences, we observe that proline occurs 351 times at the third site, then *P *("proline") = 351/2,134. Note that *P *("proline") is a conditional probability for that site variable on condition of the presumed function *f*. This is calculated for each amino acid for all sites. The functional uncertainty of the amino acids in a given site is then computed using Eqn. (1) using the estimated probabilities for each amino acid observed. The Fit value for that site is then obtained by subtracting the functional uncertainty of that site from the null state, in this case using Eqn. (4), log20. The individual Fit values for each site can be tabulated and analyzed. The summed total of the fitness values for each site can be used as an estimate for the overall FSC value for the entire protein and compared with other proteins.

### B. Measuring changes in FSC

In principle, some proteins may change from a non-functional state to a functional state gradually as their sequences change. Furthermore, iso-enzymes in some cases may catalyze the same reaction, but have different sequences. Also, certain enzymes may demonstrate variations in both their sequence and their function. Finally, a single mutation in a functional sequence can sometimes render the sequence non-functional relative to the original function. An example of this effect has been observed in experiments with the ultrabiothorax (Ubx) protein [17,33].

**Figure 1:**
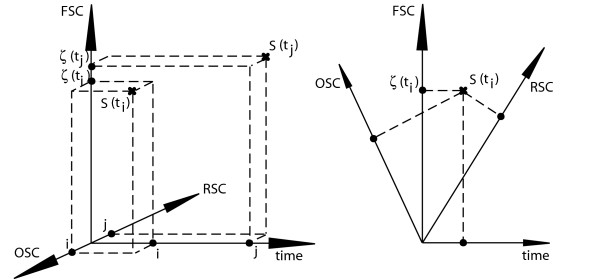
**Changing measure of FSC over time**. The measured value ζ of a biosequence S can change over time with mutation events. Changes in FSC between *t*_i _and *t*_j _may indicate changes in the amount of order or randomness of the sequence. If OSC and RSC are represented as a continuum (as shown at left), a functional sequence at time *t *will have a FSC and time value as well as OSC/RSC values. If, as shown at right, OSC, RSC, FSC and time are represented as a 4-dimensional space, then a functional sequence S(*t*_i_) will have discrete FSC, OSC, RSC and time values.

From Eqn. (6), the FSC of a biosequence can be measured as it changes with time, as shown in Figure 1. When measuring evolutionary change in terms of FSC, it is necessary to account for a change in function due to insertions, deletions, substitutions and shuffling. Evolutionary change that involves a change from a non-functional state that is not in the null state, or from an existing function *f*_a _to a modified function *f*_b _that is either different in terms of efficiency or function, is given by

ζ_E _= Δ*H *(*X*_fa_(*t*_i_), *X*_fb_(*t*_j_)).

The sequences (corresponding to *X*_fa _with initial function *f*_*a*_) have two components to it, relative to that of *X*_fb _(with resulting mutated function *f*_*b*_). The *static component *is that portion of the subsequence that must remain within the permitted sequence variation of the original biosequence with function *f*_a _while, at the same time, enabling the new function *f*_b_. The *mutating component *is the portion of *X*_fa _that must change to achieve either the new function *f*_b_, where the new function is to be understood as either a new level of efficiency for the existing function, or a novel function different from *f*_a_. This is a convenient simplification, assuming that the two components are separate according to the aligned sites. Currently we are also studying scenarios when the two components may be mixed, possibly at different times. The mutating component can be assumed to be in the null state relative to the resulting sequences of *X*_fb_. Since the mutating component is the only part that must change, we can ignore the static component *provided we include the probability of it remaining static *during the mutational changes of the mutating component.

In practice, the sequence space for possible novel functional states may not be known. However, by considering particular proteins, estimated mutation rates, population size, and time, an estimated value for the probability can be chosen and substituted into the relevant components of Eqn. (9) to limit search areas around known biosequences that are observed, such as protein structural domains, to see what other possible states within that range might have some selective advantage. In this way, possible evolutionary paths for the formation of certain protein families might be reconstructed. For example, using this method, it might be possible to predict, say, future viral strains within certain limits.

Intuitively, the greater the reduction in FSC a mutation produces, the more likely the mutation is deleterious to the given function. This can be evaluated using known mutations introduced individually into a set of aligned, wild-type sequences to measure the change in FSC. The results could then be ranked. Operating under the hypothesis that mutations producing the greatest decrease in FSC are most likely to be deleterious, experimental investigations into certain genes with certain mutations could be prioritized according to how negatively they affect FSC.

## Results and Discussion

For the 35 protein families analyzed, a measure of FSC in Fits for each site was computed from their aligned sequence data on PFAM. The results for the families, as well as an array of randomly (uniformly) generated sequences and an ordered 50-mer polyadenosine sequence are shown in Table 1. They reveal significant aspects of FSC described below.

**Table 1:** FSC of Selected Proteins

	**length (aa)**	**Number of Sequences**	**Null State (Bits)**	**FSC (Fits)**	**FSC Density Fits/aa**
Ankyrin	33	1,171	143	46	1.4
HTH 8	41	1,610	177	76	1.9
HTH 7	45	503	194	83	1.8
HTH 5	47	1,317	203	80	1.7
HTH 11	53	663	229	80	1.5
HTH 3	55	3,319	238	80	1.5
Insulin	65	419	281	156	2.4
Ubiquitin	65	2,442	281	174	2.7
Kringle domain	75	601	324	173	2.3
Phage Integr N-dom	80	785	346	123	1.5
VPR	82	2,372	359	308	3.7
RVP	95	51	411	172	1.8
Acyl-Coa dh N-dom	103	1,684	445	174	1.7
MMR HSR1	119	792	514	179	1.5
Ribosomal S12	121	603	523	359	3.0
FtsH	133	456	575	216	1.6
Ribosomal S7	149	535	644	359	2.4
P53 DNA domain	157	156	679	525	3.3
Vif	190	1,982	821	675	3.6
SRP54	196	835	847	445	2.3
Ribosomal S2	197	605	851	462	2.4
Viral helicase1	229	904	990	335	1.5
Beta-lactamase	239	1,785	1,033	336	1.4
RecA	240	1,553	1,037	832	3.5
Bac luciferase	272	1,900	1,176	357	1.3
tRNA-synt 1b	280	865	1,210	438	1.6
SecY	342	469	1,478	688	2.0
EPSP Synthase	372	1,001	1,608	688	1.9
FTHFS	390	658	1,686	1,144	2.9
DctM	407	682	1,759	724	1.8
Corona S2	445	836	1,923	1,285	2.9
Flu PB2	608	1,692	2,628	2,416	4.0
Usher	724	316	3,129	1,296	1.8
Paramyx RNA Pol	887	389	3,834	1,886	2.1
ACR Tran	949	1,141	4,102	1,650	1.7
Random sequences	1000	500	4,321	0	0
50-mer polyadenosine	50	1	0	0	0

**Results for 35 protein families **Shown above are the 35 protein families analyzed, their sequence length (column 1), the number of sequences analyzed for each family (column 2), the Shannon uncertainty of the Null State *H*_ø _(Eqn. 4) for each protein (column 3), the FSC value ζ in Fits for each protein (column 4), and the average Fit value/site (FSC/length, column 5). For comparison, the results for a set of uniformly random amino acid sequences (RSC) are shown in the second from last row, and a highly ordered, 50-mer polyadenosine sequence (OSC) in the last row. All values, except for the OSC example, which was calculated from the constrained ground state required to produce OSC, were computed from the null state. The Fit values obtained can be discussed as the measure of the change in functional uncertainty required to specify any functional sequence that falls into the given family being analyzed.

First, as observed in Table 1, although we might expect larger proteins to have a higher FSC, that is not always the case. For example, 342-residue SecY has a FSC of 688 Fits, but the smaller 240-residue RecA actually has a larger FSC of 832 Fits. The Fit density (Fits/amino acid) is, therefore, lower in SecY than in RecA. This indicates that RecA is likely more functionally complex than SecY. The results for the array of random sequences and for a 50-mer polyadenosine sequence formed on Montmorillonite show that Δ*H*_f _distinguishes FSC from RSC and OSC. The results for the array of random sequences are shown in the second from the last row of Table 1, and indicate that random sequences, which are an example of RSC, tend to have an FSC of approximately 0 Fits. The results of the highly ordered 50-mer polyadenosine, which is an example of OSC, are shown in the last row of Table 1, and indicate an FSC of approximately 0 Fits. This is consistent with Abel and Trevors' prediction that neither OSC nor RSC can contain the functional sequence complexity observed in biosequences.

**Figure 2:**
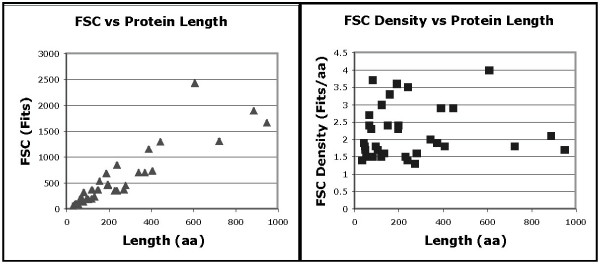
**Measuring FSC for 35 protein families**. The left plot shows the FSC with respect to the length of the aligned proteins. The x-intercept of a fitted line trace back to an approximate protein length of 23 amino acids. It also shows an absence of points in the lower right corner. The right plot shows the "density" (measure of FSC/aligned sequence length).

A plot of FSC vs. protein length for the 35 selected protein families is shown in Figure 2. The x-intercept was found to be approximately 23 amino acids. For the 35 protein families analyzed, there were no points corresponding to any protein families in the lower right area of the plot. The right hand plot in Figure 2 shows the average number of Fits/site for the 35 protein families analyzed. For our small sample of 35 protein families, we found no points between 0 and 1.3 Fits/site.

**Figure 3:**
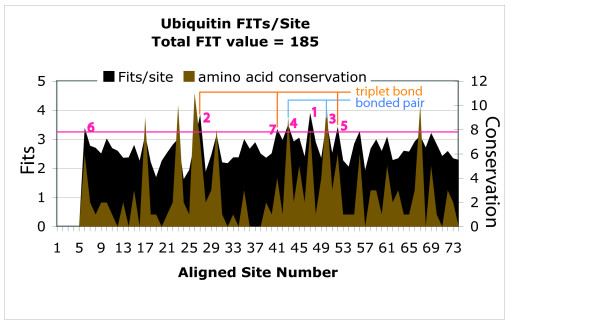
**Measure of FSC/site for ubiquitin**. The plot shows how the FSC value varies between sites for a protein family of ubiquitin. At site 37, all 20 amino acids are observed, with a relatively high Fit value of 2.9. It indicates that measure of FSC also depends on the number of amino acid types observed as well as the distribution of frequencies of each type.

To demonstrate some ways in which our approach can be applied to proteins, ubiquitin was chosen. A sample plot of FITs/site and amino acid conservation for each site, is shown in Figure 3 for Ubiquitin. Data for the first 5 sites in the aligned set was not available from PFAM. The conservation value was obtained by subtracting the total number of different options observed at a given site, from the total possible options. In this case, 20 different amino acids. If all 20 amino acids were permitted at a site, then the conservation value was 0. A maximum value of 19 would obtain if only 1 amino acid was observed at the site. From Figure 3, it can be observed that a high conservation value usually corresponded to a high measured FSC value. The measurement is also affected by the number of amino acids observed which could be different for different sites. For example, site 37 shows 20 observed amino acids, but still a relatively high value of 2.9 Fits. Conservation of a site reflects the degree of variations which is affected by both the number of observed amino acids and their frequency of observation in the alignment [12]. For example, if a site is observed to be dominated by only a few amino acids even though all the amino acid types are observed, its measured FSC could still be high.

**Figure 4:**
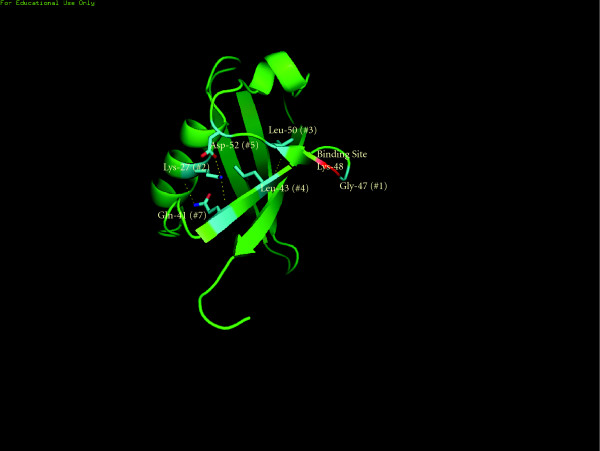
**Location of high Fit value sites for ubiquitin**. Six of the seven highest Fit value sights in ubiquitin are shown in cyan in this MacPyMOL model of 1AAR. The site with the highest Fit value in the entire protein was Gly-47, immediately next to the Lys-48 binding site, a possible indication that it plays a key role in permitting Lys-48 to undergo binding. The other 5 sites appear to perform a critical role in the conformation of the binding domain.

**Figure 5:**
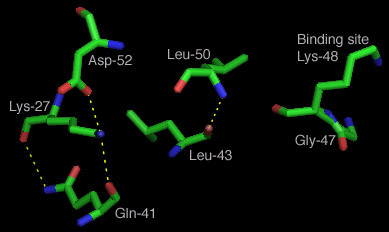
**Calculated bonds for high Fit value sites in binding domain**. A 3^rd^-order component was observed between Lys-27, Gln-41, and Asp-52 which appears to help conform the beta strand/loop leading to the binding site. The bonded pair Leu-43 and Leu-50 help conform the beta strand and loop leading away from the binding site. These 5 sites were among the 7 highest Fit value sites for ubiquitin suggesting that they play a critical role in conforming the binding domain. (Bonds were computed using the software MacPyMOL)

From the plot in Figure 3, one can observe which sites have higher measured FSC value. An arbitrary lower cutoff limit of 3.32 Fits/site was chosen to indicate high measured FSC sites, presumed to be significantly associated with the functionality of the whole molecule. A more rigorous statistical analysis can also be used as well as other types of measures based on dependency [12]. All sites exceeding that lower cutoff value were examined. A total of seven sites were found that had a high value that was between 3.32 Fits/site and the maximum of 4.32 Fits/site. These sites were located on a 3-D model of ubiquitin (1AAR.pdb) as shown in Figure 4. Out of the 7 sites, 6 where located on the binding domain containing the binding site Lys-48 [34]. The site with the maximum FIT value was site 47, immediately next to the binding site. Surprisingly, the binding site itself was poorly conserved, with an amino acid conservation value of only 1, albeit a relatively high value of 2.91 Fits. Five of the remaining 6 sites were found to be clustered in the area of the binding site, with bonds between Leu-50 and Leu-43, as well as between Gly-41, Lys-27, and Asp-52, as shown in Figure 5. Since these sites had the highest FSC values in ubiquitin, we infer that they play a critical role in either binding, or in the structure of the binding site domain for that protein. The fact that of the top seven sites, one was immediately adjacent to the binding site, and five others were located on the structure supporting the binding site lends support for our hypothesis that high FIT values can be used to locate key functional components of a protein family.

**Figure 6:**
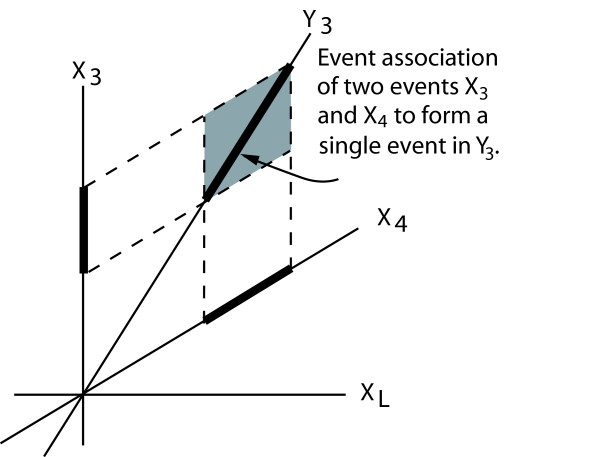
**Detecting higher order dependencies**. A biosequence of *L *sites can be represented as an *L*-discrete space *X*_L_. A pairwise relation between sites 3 and 4 can be represented as a single event at site 3 in an *R*-discrete space *Y*_R_. The FSC value computed after the aligned sequence data has been converted to *Y*_R _would yield a more accurate measurement of FSC.

In this paper, we have presented an important advance in the measurement of the FSC of biopolymers. It was assumed that aligned sequences from the same Pfam family, could be assigned the same functionality label. Even though the same functionality may not be applicable to individual sites, site independence and significance was assumed and the measured FSC of each site was summed. However, further extension of the method should be considered [12,35]. For example, if dependency of joint occurrences is detected between the outcomes of two variables *X*_3 _and *X*_4 _in the aligned sequences, then the N-tuple representation of the sequences could be transformed into a new R-tuple *Y*_R _where these outcomes of *X*_3 _and *X*_4 _are represented as outcome by a single variable *Y*_3 _as shown in Figure 6. An outcome of the two variables in *X*_3 _and *X*_4 _correspond to a hypercell in Y_R_. A more accurate estimate of FSC could then be calculated. We are currently considering this more general scenario.

The measurement in Fits of the FSC provides significant information about how specific each monomer in the sequence must be to provide the needed/normal biofunction. The functional information measures the degree of challenge involved in searching the sequence space for a sequence capable of performing the function. In addition, Fits can be summed for every sequence required to achieve a complete functional biochemical pathway and integrated cellular metabolism, including regulatory proteins. In principle, it may be possible to estimate a FSC value for an entire prokaryotic cell where the genome has been sequenced and all translated proteins are known. Simpler genomes in viruses may be an excellent example for this kind of analysis. That is, further analysis of the FSC values will provide a starting point to reveal important information about the processes for an entire organism such as the virus.

## Conclusion

A mathematical measure of functional information, in units of Fits, of the functional sequence complexity observed in protein family biosequences has been designed and evaluated. This measure has been applied to diverse protein families to obtain estimates of their FSC. The Fit values we calculated ranged from 0, which describes no functional sequence complexity, to as high as 2,400 that described the transition to functional complexity. This method successfully distinguishes between FSC and OSC, RSC, thus, distinguishing between order, randomness, and biological function.

## Methods

The following is a brief summary of the methods used (additional file 1). A more detailed description is available as an online supplement. Eqn. (6) was applied to 35 protein families or protein domain families, to estimate the value of the FSC for any protein included within that family. A program was written, using Python, to analyze the two-dimensional array of aligned sequences for a protein family and is available online (additional files 2, 3, 4, 5, 6, 7, 8, and 9).  The data for the arrays was obtained from the Pfam database [36]. Eqn. (7) was used to estimate the probability for each amino acid at each site.

## Competing interests

The author(s) declare that they have no competing interests.

## Authors' contributions

KD helped develop the formulation and measure of FSC, wrote the software, carried out the analysis and drafted the manuscript. DC developed, together with KD, the basic formula of functional entropy and evaluated the different mathematical forms. DC provided suggestions to the experimental design and its interpretations. DLA's contributions included defining/delineating the three subsets of sequence complexity and their relevance to biopolymeric information, contributing to the first draft of the paper, critiquing KD's quantification methodology, contributing references, and coining the term "fits" for "functional bits" as the unit of measure of Functional Sequence Complexity (FSC). JT participated in the design and coordination of the study. All authors read and approved the final manuscript.

## Supplementary Material

Additional File 1 Methods. Additional details of the methods used in this project Click here for file

Additional File 2 Main Program. A copy of the coding for the main program used in this paper Click here for file

Additional File 3 AminoFreq. A required module for the main program Click here for file

Additional File 4 ColTot. A required module for the main program Click here for file

Additional File 5 Convert. A required module for the main program Click here for file

Additional File 6 DistEnt. A required module for the main program Click here for file

Additional File 7 FormArray. A required module for the main program Click here for file

Additional File 8 StripName. A required module for the main program Click here for file

Additional File 9 P53DNADom. A sample data set for the reader Click here for file
