# Supplementary material for: Measuring the functional sequence complexity of proteins
Source: Theor Biol Med Model. 2007 Dec 6;4:47. doi: 10.1186/1742-4682-4-47 (PMC2217542; doi:10.1186/1742-4682-4-47)
Supplement: Additional File 1 — Methods. Additional details of the methods used in this project [file 1742-4682-4-47-S1.rtf]

Measuring the Functional Sequence Complexity of proteins MethodsEqn. (6) was applied to 35 proteins or protein domains to estimate the value of the FSC for a protein included in that family. A program was written, using Python, to analyze the two-dimensional array of aligned sequences for a protein family and is available at http://www.uoguelph.ca/~kdurston/. The data for the arrays was obtained from the Pfam database (http://pfam.wustl.edu/). The length of each sequence, including identifiers, and the number of sequences in the array were input into the program. The two-dimensional aligned sequence array for a particular protein was read by the program and a computational analysis was performed. The entire array of aligned sequences was treated as a single, unified message that described the functional sequence space for a protein family.The ∆ for each column in the array was computed by moving through each column in the set of aligned sequences and compiling the number of occurrences of each amino acid in each column. The estimated probability P for each amino acid represented in the column was equal to the number of occurrences of that amino acid in that column divided by the total number of aligned sequences in the set. There are numerous columns in a flat set of aligned sequences that contain insertions or deletions. These are usually in regions that have considerable flexibility and may, or may not contribute to the functional complexity of the protein. To avoid the effect of the columns representing indels, a cutoff value was input into the program. The cutoff value represented the minimum number of amino acids occurring in a column divided by the total number of aligned sequences. If the total number of amino acids in a given column was below the cut-off value, due to a large number of indel-produced vacancies in the column, then the value of ∆ was automatically set to zero. Since these regions indicate little or no sequence conservation, they are already close to the null state, so setting ∆ = 0 is a reasonable move and prevents such columns from a spurious contribution to the overall FSC of the protein. The cutoff value was adjusted from a minimum of .55 to a maximum of .89 such that the number of remaining sites to be evaluated was very close to the standard protein length suggested by Pfam. The total number of remaining sites was output as the size of the protein. The ∆ for each column was summed and then output as the estimation in Fits of the FSC of the protein. The FSC density of each protein was computed by dividing the estimated number of Fits by the size N of the protein. 	To illustrate the FSC of sequences that only had RSC, an array of 500 uniformly random sequences were generated, each having 1000 sites. The array was input into the software to compute the value in Fits of the FSC of the set of random sequences. To illustrate OSC, the Fit value of a 50-mer sequence of polyadenosine produced on Montmorillonite clay was calculated according to Eqn. (2), where Xg represented the ground state. The calculation, however, was trivial since the ground state for the physical constraints permits only one repeating pattern. 	
