# Supplementary material for: Measuring the functional sequence complexity of proteins
Source: Theor Biol Med Model. 2007 Dec 6;4:47. doi: 10.1186/1742-4682-4-47 (PMC2217542; doi:10.1186/1742-4682-4-47)
Supplement: Additional File 2 — Main Program. A copy of the coding for the main program used in this paper [file 1742-4682-4-47-S2.doc]

MEASURING THE FUNCTIONAL SEQUENCE COMPLEXITY OF PROTEINS

Included below is the main program and its required modules. The programing language is Python. The program is currently configured to analyze the p53 DNA binding domain as an example. The data file for this domain is included below the modules.

To use the program, click on each of the links below, then copy and paste the content of each into individual python windows. Each window should be saved with the title used in the link (e.g., FormArray.py). To verify that everything is working properly, it is recommended that once the user has downloaded and installed all the program files, the data for the p53 DNA binding domain should be run to see if the same results are obtained as in the paper (FSC = 525, active sites = 157, etc.). Once the same results are obtained, then the program is ready for use.

To evaluate a particular protein, a set of aligned sequences should be converted into one long string and saved as a Python readable file. To run the program for that protein, the following inputs will be necessary:

Length: (the total length of the sequences in the alignment, including the sequence identifiers)

Number: (the number of sequences in the alignment)

NameLength: (the length of the sequence identifiers at the front of each sequence in the alignment ... if no identifier is used, then this value is 0)

input: (the name of the data file should be input within the single qoutes ... e.g. open('P53DNADom','r') for the p53 data)

Cutoff: (For any column in the set of alignment, there will often be spaces due to insertions elsewhere in the alignment. The total number of amino acids in a column divided by the total number of sequences is compared to the cutoff value, which ranges between 0.1 and 1.0. If the number of amino acids in that column falls below cutoff value that is input, then the FSC of that column is set to zero. This procedure is to eliminate possible spurious contributions by insertions that are not likely to have a function in the protein. For this reason, the cuttoff value should be chosen with care. One method is to chose the cuttoff value such that the number of active sites is approximately equal to the generally accepted length of that protein or protein domain.)

For each protein analyzed, the individual site FSCs are given so that they can be plotted and a more detailed picture of the FSC for that protein can be obtained.

The main program is as follows:

import FormArray

import StripName

import AminoFreq1

import ColTot

import DistEnt

import Convert

import math

Length = 265 #input length of protein sequence

Number= 156 #input number of alignments

NameLength=21 #input number of spaces taken up by protein sequence ID

NumPossibilities=20 #input number of possibilities per locus, 20 for amino acids, 4 for bases

input = open('P53DNADom','r') #open file of alignments, file should be one long string

Cutoff=0.75 #weeds out occasional insertions

LongString = input.readline() #read alignments in file

input.close()

LongList = list(LongString)

NumSites= Length-NameLength

MinCut=int(Number*Cutoff)

#CONVERT LongList INTO AN ARRAY OF INDIVIDUAL SEQUENCES

Array=[]

Array= FormArray.formarray(Array, Number, Length, LongList)

#STRIP OFF PROTEIN IDENTIFIER FOR EACH PROTEIN SEQUENCE

SeqArray=[]

SeqArray=StripName.stripname(SeqArray, Number, Array, NameLength)

#This section determines the number of occurances of each amino acid at each site

AminoAcids = ['_','G','A', 'V', 'L','I','M','F','W','P','S','T','C','Y','N','Q','D','E','K','R','H']

NumOptions=len(AminoAcids)

Occurances = AminoFreq1.aminofrequency(NumSites, Number, SeqArray, AminoAcids)

#THIS SECTION PRINTS THE OCCURANCES IF RESULTS IS SET TO 'yes'

results = 'no'

if results == 'yes':

print 'Occurances'

n=0

while n<21:

temp=Occurances[n]

print temp

n+=1

#THIS SECTION COMPUTES THE TOTAL NUMBER OF SEQUENCES PER COLUMN THAT HAVE ACTIVE AMINO ACIDS

ColumnTotals, ActiveSites = ColTot.totals(NumOptions, NumSites, Occurances, MinCut)

#THIS SECTION COMPUTES THE FUNCTIONAL ENTROPY FOR EACH COLUMN IN EACH PASS

TotalDistEnt, DistEntropies=DistEnt.distentropies(NumOptions, NumSites, Occurances, ColumnTotals, MinCut)

#This section coverts from log10 to Fits if Fits is set to 'yes'

Fits='yes'

if Fits=='yes':

TotalDistEnt, DistEntropies=Convert.conversion(TotalDistEnt, DistEntropies, NumSites)

#COMPUTE FUNCTIONAL ENTROPY PER ACTIVE SITE

Density=TotalDistEnt/ActiveSites

#COMPUTE STANDARD SHANNON UNCERTAINTY

Shannon=float((ActiveSites*math.log10(20))/math.log10(2))

print 'Were results converted to Fits?', Fits

print' '

print 'Distribution ∆Hf by column is:'

print DistEntropies

print' '

print 'Number of sites with amino acid occurances equal to or greater than MinCut =', ActiveSites

print' '

print 'Total ∆Hf if frequency of occurance/site is used =', "%.0f"%TotalDistEnt

print' '

print 'Shannon uncertainty =', "%.3f"%Shannon

print' '

print 'FC density out of a maximum of 4.32 if Fits is used, or 1.301 if Fits is not used =', "%.2f" %Density

print' '

print 'End of results'
