# Supplementary material for: Measuring the functional sequence complexity of proteins
Source: Theor Biol Med Model. 2007 Dec 6;4:47. doi: 10.1186/1742-4682-4-47 (PMC2217542; doi:10.1186/1742-4682-4-47)
Supplement: Additional File 3 — AminoFreq. A required module for the main program [file 1742-4682-4-47-S3.doc]

def aminofrequency(length, number, array, aminoacids):

finalarray = list(aminoacids)

site = 0

while site<length: #search through each site in the array

tempa=list(finalarray[0])

tempa.append(site+1)

finalarray[0]= tempa

aa=1

while aa<21: #work through each amino acid

n = 0

tempa=list(finalarray[aa])

counter = 0

while n < number:

if array [n][site] == aminoacids[aa]:

counter +=1

n+=1

tempa.append(counter)

finalarray[aa] = tempa

aa+=1

site+=1

return finalarray
