# Supplementary material for: Measuring the functional sequence complexity of proteins
Source: Theor Biol Med Model. 2007 Dec 6;4:47. doi: 10.1186/1742-4682-4-47 (PMC2217542; doi:10.1186/1742-4682-4-47)
Supplement: Additional File 4 — ColTot. A required module for the main program [file 1742-4682-4-47-S4.doc]

def totals(numoptions, numsites, occurances, mincut):

columntotals=[]

UpCut=mincut-1

activesites=0

n=1

length=numsites+1

while n<length:

m=1

ColumnTotal = 0

while m<numoptions:

temp=occurances[m][n]

ColumnTotal=ColumnTotal+temp

m+=1

Denom=float(ColumnTotal)

columntotals.append(Denom)

n+=1

if Denom>UpCut:activesites+=1

return columntotals, activesites
