# Supplementary material for: Measuring the functional sequence complexity of proteins
Source: Theor Biol Med Model. 2007 Dec 6;4:47. doi: 10.1186/1742-4682-4-47 (PMC2217542; doi:10.1186/1742-4682-4-47)
Supplement: Additional File 5 — Convert. A required module for the main program [file 1742-4682-4-47-S5.doc]

def conversion(totaldistent, distentropies, numsites):

import math

denominator=math.log10(2)

totaldistent=totaldistent/denominator

n=0

while n<numsites:

temp=float(distentropies[n])

fittemp=temp/denominator

fittemp="%.2f"%fittemp

distentropies[n]=fittemp

n+=1

return totaldistent, distentropies
