# Supplementary material for: Measuring the functional sequence complexity of proteins
Source: Theor Biol Med Model. 2007 Dec 6;4:47. doi: 10.1186/1742-4682-4-47 (PMC2217542; doi:10.1186/1742-4682-4-47)
Supplement: Additional File 6 — DistEnt. A required module for the main program [file 1742-4682-4-47-S6.doc]

def distentropies(numoptions, numsites, occurances, columntotals, mincut):

import math

Total=0

Results=[]

# Compute total occurances for each column

n=1

times=numsites+1

while n<times:

Samples=float(columntotals[n-1])

m=1

ColumnEntropy=0

while m<numoptions:

if Samples<1:break

else:

temp=float(occurances[m][n])

if temp<1:FuncSiteEntropy=0

else:FuncSiteEntropy=-(temp/Samples*math.log10(temp/Samples))

ColumnEntropy=ColumnEntropy+FuncSiteEntropy

m+=1

if Samples<mincut:DeltaColumnEntropy=0

else:DeltaColumnEntropy=math.log10(20)-ColumnEntropy

Results.append("%.3f" %DeltaColumnEntropy)

Total = Total + DeltaColumnEntropy

n+=1

return Total, Results
