# Supplementary material for: Measuring the functional sequence complexity of proteins
Source: Theor Biol Med Model. 2007 Dec 6;4:47. doi: 10.1186/1742-4682-4-47 (PMC2217542; doi:10.1186/1742-4682-4-47)
Supplement: Additional File 7 — FormArray. A required module for the main program [file 1742-4682-4-47-S7.doc]

def formarray(array, number, length, longlist):

loc=0

n=0

while n<number:

protein=[]

site = 0

while site<length:

temp = longlist[loc]

protein.append(temp)

loc+=1

site+=1

array.append(protein)

n+=1

return array
