# Supplementary material for: Measuring the functional sequence complexity of proteins
Source: Theor Biol Med Model. 2007 Dec 6;4:47. doi: 10.1186/1742-4682-4-47 (PMC2217542; doi:10.1186/1742-4682-4-47)
Supplement: Additional File 8 — StripName. A required module for the main program [file 1742-4682-4-47-S8.doc]

def stripname(seqarray, number, array, namelength):

n=0

seqarray=[]

while n<number:

member=[]

member=array[n]

temp=member[namelength:]

seqarray.append(temp)

n+=1

return seqarray
